# Regeneration of Spent Desiccants with Supercritical CO_2_

**DOI:** 10.1021/acs.iecr.4c03636

**Published:** 2024-11-27

**Authors:** Astrid Melissa Rojas Márquez, Iris Beatriz Vega Erramuspe, Brian K. Via, Bhima Sastri, Sujit Banerjee

**Affiliations:** a Forest Products Development Center, Auburn University, Auburn, Alabama 36849, United States; b US Department of Energy, 19901 Germantown Road, Germantown, Maryland 20874, United States; c School of Chemical & Biomolecular Engineering, Georgia Tech, Atlanta, Georgia 30332, United States

## Abstract

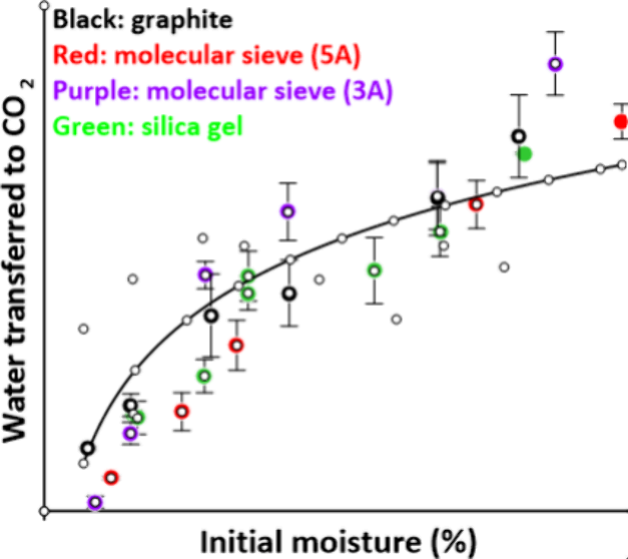

Supercritical CO_2_ (sCO_2_) dehydrates desiccants such as silica gel, activated carbon, graphite, and molecular sieve by dissolving and emulsifying the water. Despite differences in the surface area of these desiccants, the amount of water removed under comparable conditions is the same. The main advantage of sCO_2_ dewatering over conventional hot-air regeneration lies in situations where the exhaust contains environmentally sensitive components, e.g., in nuclear detritiation operations where the small footprint and closed cycle benefits of the sCO_2_ process are especially significant. Calculations show that depressurizing the spent sCO_2_ to half its initial pressure drops out most of the water, after which the CO_2_ can be repressurized and reused. sCO_2_ dewatering requires about half the energy needed for thermal drying because the water is removed nonevaporatively.

## Introduction

Desiccants used in industry are typically dehydrated through temperature swing adsorption. High temperatures of 200–250 °C are required for molecular sieve;^1,2^ less aggressive conditions suffice for dessicants such as silica gel^3^, where solar dryers can even be used. The energy burden for regeneration is high because the water is removed evaporatively. Supercritical CO_2_ (sCO_2_) has recently been used to dewater a wide range of materials ranging from ion exchange resins^4^ to sludge.^5^ The water is both dissolved and emulsified in the sCO_2_^6^, which can then be partially expanded to release the water. The energy savings are considerable because the water is removed nonevaporatively at 90 °C. sCO_2_ has also been used to decontaminate spent sorbents such as activated carbon from compounds such as chlorophenol^7^ and xylene.^8^ While these spent sorbents are frequently water-laden, especially when they are used to remove dissolved contaminants from water, the focus has been on removing the organic contaminants from the sorbent rather than on dewatering it. This paper describes the removal of water by sCO_2_ from molecular sieve, activated carbon, graphite and silica gel, and interprets the different conditions that appy to each desiccant. Because use of sCO_2_ requires a pressure vessel, the approach is especially suitable for high-value low-volume applications, such as the regeneration of desiccants used to capture tritiated water vapor in the nuclear industry.^2,9^

## Experimental Section

Graphite powder (<5 μm) from ChemicalStore.com, acid-washed granular activated carbon (0.2–5 mm) from Calgon, silica gel (40–63 μm) from Millipore-Sigma, and molecular sieve (3A and 5A zeolite) from Wisesorbent Technology were used in this study. BET surface area measurements were made with a Micromeritics TriStar II Plus analyzer.

The supercritical CO_2_ extractor used was a Super C unit from OCO Laboratories. The procedure for sCO_2_ treatment is straightforward and has been described in detail earlier.^4^ Briefly, the desiccant/water mixture was placed in an aluminum boat (∼8 mL capacity) and contacted (batch mode) with sCO_2_ in a 120-ml chamber. The instrument continuously adjusts temperature and pressure to keep these variables within the preset values for each extraction. Following decompression of CO_2_, the sample was cooled to room temperature in a desiccator and weighed, with the weight loss being attributed to the water removed.

The sCO_2_: desiccant mass ratio was obtained by calculating the volume of the desiccant from its specific gravity. This volume was subtracted from the extractor volume of 120 mL. The remaining reactor volume was assumed to be occupied by sCO_2_ whose density and mass were determined at the corresponding temperature and pressure from the equation of state of Span and Wagner.^10^ Most of the measurements were made at 90 °C and 8.3 MPa with an exposure time of 20 min. The mole fraction concentration of water in sCO_2_ (*x*) rises with increasing temperature, a trend that is consistent with all our previous work^4−6,11^ on a variety of substrates. Pressure has a smaller effect on *x*. For example, *x* values for dewatering graphite obtained from runs conducted at 90 °C for 20 min at 8.3 and at 11.0 MPa were statistically identical, because of the similar solubilities of water in sCO_2_ at these two pressures.^12^ The value of *x* increases with increasing exposure time, but plateaus at about 20 min, which matches our previous result obtained with several other matrices.^4,11^

## Results and Discussion

**Figure 1:**
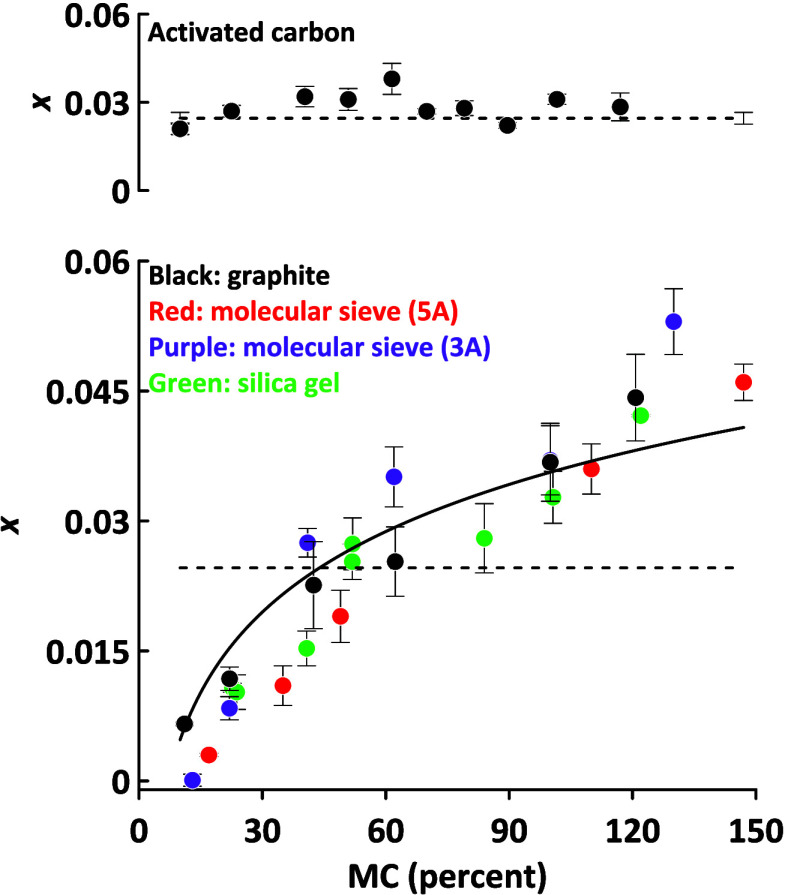
sCO_2_ dewatering of graphite, molecular sieves 3A and 5A, and silica gel at 90 °C and 8.3 MPa (lower panel) and activated carbon (upper panel). The dashed line represents the solubility of water in sCO_2_.

Previous work identified three mechanisms of water transfer from solids to sCO_2_.^6^ First, if the water is tightly bound to the solid its concentration in sCO_2_ is well below its solubility limit. Second, when the water is less strongly bound (as in some sludges) the concentration, *x*, is at the solubility limit. Third, if there is an excess of water, it can be emulsified in sCO_2_, and its effective concentration in sCO_2_ can rise well above the solubility limit. Results from the dewatering of graphite, molecular sieve (3A and 5A) and silica gel are illustrated in lower panel of Figure 1, where *x* is plotted against the initial dry basis (water/dry solids) moisture content (MC). Corresponding values for activated carbon are plotted separately in the upper panel of Figure 1 for the sake of clarity. The dashed line is the solubility of water in sCO_2_ using the value of *x* = 0.024 reported by Wang et al.^12^ Because the high MCs in Figure 1 exceed the saturation levels of the desiccants they will not be reached in a practical setting; the *x* values taken at these levels are only provided to relate them to the water solubility line. The curves for the desiccants in Figure 1 fall below the solubility line at low MC but rise above it at higher MC levels. We have noted similar behavior during the dewatering of Amberlyst resin.^4^

The transfer of water to sCO_2_ is controlled by the sCO_2_:desiccant distribution coefficient of water. If the water is strongly bound to the desiccant, then its transfer to sCO_2_ will be suppressed. At intermediate MC levels, the desiccant sites that strongly attract water will be saturated and water transfer to sCO_2_ will be governed by its solubility. Values of *x* at high MC exceed the solubility line because of the onset of emulsification, even without active agitation. In previous work we found that the emulsified water in sCO_2_ reached a nominal mole fraction concentration of over 0.1.^4^ The surface areas of the desiccants used are listed in Table 1 and are related to pore size. Surprisingly, they do not influence the *x* values in Figure 1. Except for activated curves all the curves in Figure 1 are similar under high moisture conditions, because the dominant factor is the emulsification of water in sCO_2_. The profile for activated carbon is flat across the various MC values. The reason for this anomalous behavior is unknown, but the properties of activated carbon are very different from the others in that it is much more hydrophobic with a much smaller surface area and a larger particle size.

**Table 1:** Nitrogen BET Surface Areas of Desiccants

	BET surface area (m^2^/g)
graphite	9.86
silica gel	476
activated carbon	1.92
molecular sieve 3A	26^a^
molecular sieve 5A	457

a ^1^ From ref (13).

**Figure 2:**
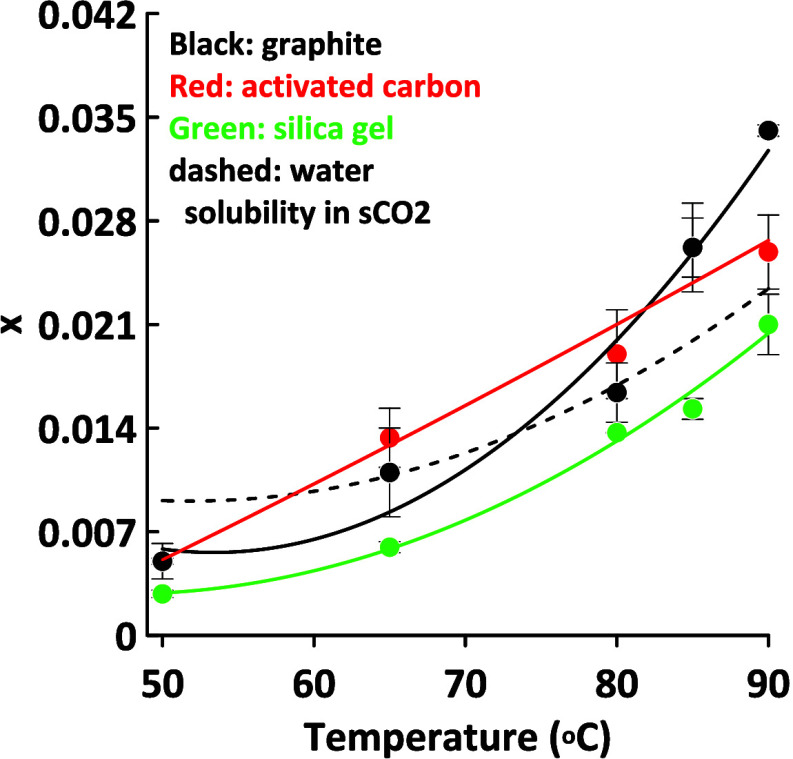
Temperature dependence of sCO_2_ dewatering of activated carbon, graphite and silica gel at 8.3 MPa and ∼65% MC. The dashed line represents the solubility of water in sCO_2_.

The temperature dependencies of *x* are shown in Figure 2 for several desiccants under a common set of extraction conditions (90C, 8.3 MPa, 20 min). All three desiccants show similar curves, but their behavior vis-à-vis the water solubility line is markedly different. For all three desiccants, *x* is lower than the water solubility line at low temperatures, but only the graphite and activated carbon curves rise above it beyond 80 °C. Clearly the strength of water binding to the desiccant is strongly temperature dependent. The curve for activated carbon is similar but not identical to the water solubility curve. However, these differences are relatively small; overall, water solubility is clearly the controlling factor.

Following extraction, the sCO_2_ will need to be expanded to release the entrained water so that the CO_2_ can be compressed for reuse. As the sCO_2_ cools on expansion the solubility of water in sCO_2_ drops correspondingly. Consider a situation where water-laden sCO_2_ is expanded adiabatically from 90 °C to the two subcritical conditions listed in Table 2. The enthalpies listed in Table 2 were obtained^14^ with the assumption that the adiabatic expansion is isentropic. The amount of water (dissolved and emulsified) carried out by the sCO_2_ under these conditions was measured and is included in Table 2. We note that at 8.3 MPa, *x* is much higher than the solubility limit of 0.024 at 8.3 MPa and 90 °C; the difference represents emulsified water. Hence, if the sCO_2_ was expanded from 8.3 to 4.1 MPa, *x* would drop by 0.053 or 77%; this difference represents the amount of water that could be removed from the system with a cyclone or other separator. The energy required to recompress the sCO_2_ to its initial value is the enthalpy difference between the two states, i.e. Twenty-four kJ/kg per cycle. The difference in *x* of 0.053 corresponds to a CO_2_/water mass ratio of 46. Hence, 46 kg of sCO_2_ would be needed to remove 1 kg of water, at an energy cost of 1,100 kJ (from Table 2), which is about half of the value of 2,260 kJ/kg required for evaporation. Clearly, the energy savings from the nonevaporative water removal pathway offered by the sCO_2_ process is considerable. These calculations are an approximation because they do not take into account process inefficiencies, which will increase the enthalpy difference for both evaporative and sCO_2_ processes. Also, the 2,260 kJ/kg estimate for water evaporation underestimates the actual value because the bound water will resist evaporation. For example, Golubovic et al. have shown that for molecular sieves, the heat of sorption can be up to 50% greater than the latent heat of vaporization^15^, which would make the sCO_2_ even more energy cost competitive. Finally, because the sCO_2_ process is self-contained, the only waste release will be the extracted water, as opposed to thermal drying, where the volume of air emissions will be orders of magnitude higher.

**Table 2:** Isentropic Expansion of sCO_2_

pressure (MPa)	temperature (°C)	enthalpy (kJ/kg)	x
8.3	90	503	0.07 ± 0.01
6.2	72	497	0.055 ± 0.01
4.1	40	479	0.017 ± 0.004

In conclusion, we have demonstrated that sCO_2_ extraction is a viable process for reconditioning desiccants. Because of the high capital costs associated with pressure vessels, the approach is unsuitable for low-value high-volume applications where the extracted water is directly exhausted to the atmosphere, *e.g*. where desiccant wheels are used. The main advantage of sCO_2_ dewatering lies in situations where the exhaust contains components that must be collected and treated, where the small footprint and closed cycle benefits of the process are considerable. A potential application lies in the detritiation units used in nuclear plants^16^ where the radioactive water needs to be contained in as small a volume as possible. Also, the relatively mild temperatures used for sCO_2_ regeneration should increase the life of the desiccant when compared to high temperature swing operations where the desiccants physically degrade and lose their adsorption capacity with increasing temperature.^17^ While the work described here was run in a batch mode, procedures for continuous operations are also available.^18^ The pressure requirement of 8.3 MPa is just above the supercritical pressure of CO_2_ of 7.4 MPa. Commercial supercritical reactors are usually built to accommodate much higher pressures, so there is an opportunity for capital cost savings. Ultimately, the commercial feasibility of our approach will depend on the cost of both capital and of energy as well as throughput.
